# Germacranes and *m*-Menthane from *Illicium lanceolatum*

**DOI:** 10.3390/molecules19044326

**Published:** 2014-04-04

**Authors:** Ming Zhao, Xianming Zhang, Yan Wang, Min Huang, Jin-Ao Duan, Tanja Gödecke, Karina M. Szymulanska-Ramamurthy, Zhiqi Yin, Chun-Tao Che

**Affiliations:** 1 Department of Medicinal Chemistry and Pharmacognosy, College of Pharmacy, University of Illinois at Chicago, Chicago, IL 60612, USA; E-Mails: tan13ja@gmail.com (T.G.); kszymu2@uic.edu (K.M.S.-R.); chyzq2005@126.com (Z.Y.); chect@uic.edu (C.-T.C.); 2 WHO Collaborative Center for Traditional Medicine, College of Pharmacy, University of Illinois at Chicago, Chicago, IL 60612, USA; 3 Department of Pharmacology, College of Medicine, University of Illinois at Chicago, Chicago, IL 60612, USA; E-Mail: zhangxm1973@gmail.com; 4 State Key Laboratory of Bioactive Substance and Function of Natural Medicines, Institute of Materia Medica, Chinese Academy of Medical Science and Peking Union Medical College, Beijing 100050, China; E-Mail: Huangmin@imm.ac.cn; 5 Jiangsu Key Laboratory for TCM Formulae Research, Nanjing University of Traditional Chinese Medicine, Nanjing 210046, China; E-Mail: Duanja@163.com; 6 Department of Natural Medicinal Chemistry, China Pharmaceutical University, Nanjing 210009, China

**Keywords:** *Illicium lanceolatum*, Illiciaceae, germacrane sesquiterpenes, *m*-menthane monoterpene, absolute stereochemistry, proliferative promotion, SH-SY5Y

## Abstract

Three new germacrane sesquiterpenes and a new *m*-menthane monoterpene were isolated together with four known compounds from the pericarp of *Illicium lanceolatum*, an adulterant to star anise (*Illicium verum*). All compounds were isolated from *Illicium* plants for the first time. The absolute stereochemistry of all germacranes and *m*-menthane was established by a combination of NMR and the modified Mosher’s ester method. The biological activity was evaluated on SH-SY5Y neuroblastoma cell line. (1*S*,5*R*,7*R*)-1,5-Dihydroxygermacra-4(15),10(14),11(12)-triene (at 62.5 µM) and (1*R*,5*R*,7*R*)-1,5-dihydroxygermacra-4(15),10(14),11(12)-triene (at 15.6 µM) promoted the proliferation of SH-SY5Y by 36.2% and 45.8%, respectively, after 48 h incubation, indicating potential neurotrophic activity.

## 1. Introduction

The genus *Illicium* L. (Illiciaceae) consists of *ca*. 40 species that form one of the earliest evolutionary branches of the angiosperms [1]. This small taxon is represented by evergreen trees and shrubs disjunctively distributed in North America, Mexico, Peru, the West Indies and eastern Asia, with the highest concentration of species found in northern Myanmar and southern China [1,2]. The most well-known member of this genus is probably *Illicium verum*. It serves as the source material of shikimic acid in the production of oseltamivir (Tamiflu) [3], and its ripe pericarps (known as star anise) are widely used as a spice in many countries in Asia, in particular, China, India, and Vietnam [4]. *I. verum* also has a long history of medicinal applications in China [5]. In Mexico and the southwestern United States, its fruits are used to make herbal tea to alleviate colic of babies and stomach aches [4,6]. However, in recent years, intoxication cases related to the culinary and medicinal use of star anise have been reported, associating with neurological effects such as seizures, vomiting, jitteriness, rapid eye movement, and even death [7,8,9]. Follow-up investigations indicated that most, if not all, of the adverse effects were caused by adulterated toxic *Illicium* plants. Phytochemical and biological studies pointed to *seco*-prezizaane sesquiterpenes (such as anisatin and neoanisatin) to be the toxic ingredients [10,11,12]. The toxicological mechanism was elucidated to be a picrotoxin-like, non-competitive antagonism to the γ-aminobutyric acid (GABA) receptor [13,14,15,16]. However, systematic studies of the structure-toxicity relationship are limited [17,18,19]. To safeguard the use of star anise and its products, studies on adulterant species of *Illicium* is warranted. 

Apart from the potential toxicity, some ingredients of *Illicium* plants are known to display neurotrophic properties. Among others, jiadifenin, jiadifenolide, illicinin A, and 4-allyl-2,6-dimethoxy-3-(3-methylbut-2-enyl)phenol have been reported to promote neurite outgrowth in primary cultures of fetal rat cortical neurons [20,21,22,23]. *Illicium* plants are thus considered a potential source of neurotrophin-like natural products. 

We are interested in constructing a library of secondary metabolites of *Illicium* plants, to identify toxic components on one hand, and search for neurotrophin-mimic natural products on the other. Several *Illicium* species are being investigated in our group. As part of the studies, *I. lanceolatum*, a toxic adulterant of Chinese star anise, was investigated for its chemical composition. This paper reports the structures of four germacrane sesquiterpenes (including three new structures), a new *m*-menthane monoterpene, and three other known compounds, and their biological activities in the SH-SY5Y neuroblastoma cell line.

## 2. Results and Discussion

**Figure 1:**
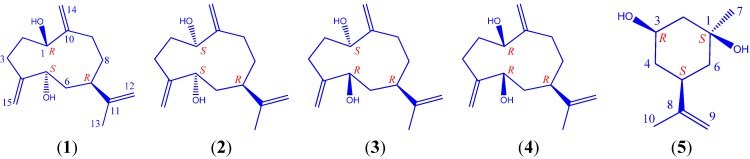
Structures of compounds **1**–**5**.

From the pericarps of *I. lanceolatum*, repeated open column (silica gel, RP-18, MCI, and Sephadex LH-20) and semi-preparative chromatographic separations resulted in the purification of four germacrane sesquiterpenes **1**–**4**, and a *m*-menthane monoterpene **5** (Figure 1), together with three other known compounds **6**–**8**. Germacrane **4** was a known compound, but its absolute stereochemistry was newly established in the present work.

Compound **1** was obtained as a white amorphous powder. A molecular formula of C_15_H_24_O_2_ was determined based on the HR-ESI-MS result at *m/z* 219.1733 [M−H_2_O+H]^+^ (calcd. 219.1743), indicating four degrees of unsaturation. The ^1^H-, ^13^C- and DEPT-NMR spectra (Table 1 and Table 2) indicated the presence of one methyl, eight methylenes (including three olefinic ones), three methines (including two oxygenated groups), and three quaternary olefinc carbons. The presence of three double bonds accounted for three degrees of unsaturation, the remaining one was therefore deduced arising from a ring structure in the molecule.

**Figure 2:**
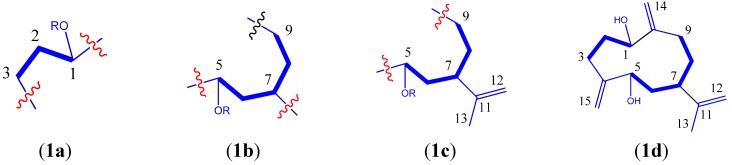
Partial structures of **1**.

**Figure 3:**
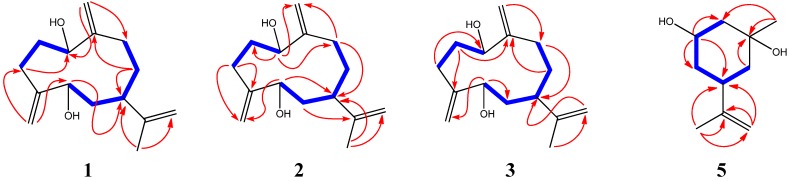
Selected ^1^H-^1^H COSY (**—**) and HMBC (**→**) correlations of compounds **1**–**3**, **5**.

In the ^1^H-^1^H COSY spectrum, signals at *δ*_H_ 1.98 and 2.10 (*m*, H-2) displayed correlations with signals at *δ*_H_ 4.24 (dd, *J* = 5.2, 10.5 Hz, H-1) and *δ*_H_ 2.29 (m, H-3), respectively, suggesting the presence of structural fragment **1a** (Figure 2). The correlations of* δ*_H_ 1.83 (m, H-6) with *δ*_H_ 4.36 (dd, *J* = 3.9, 7.0 Hz, H-5) and *δ*_H_ 2.68 (m, H-7), and that of *δ*_H_ 1.68 (m, H-8) with *δ*_H_ 2.68 (m, H-7) and *δ*_H_ 2.04 (m, H-9), led to the establishment of fragment **1b** (Figure 2). In the HMBC (Figure 3), the cross peaks between *δ*_H_ 1.76 (s, H-13) and *δ*_C_ 39.7 (CH, C-7), *δ*_C_ 151.3 (C, C-11) and *δ*_C_ 110.3 (CH_2_, C-12) suggested a partial structure **1c** (Figure 2). The connection of **1a** and **1c** via two exocyclic double bonds was established based on the following evidence: long-range correlations between *δ*_H_ 5.16 (s, H-14) and 77.2 (CH, C-1) and 29.8 (CH_2_, C-9); as well as those between *δ*_H_ 5.13/5.02 (both s, H-15) and 72.5 (CH, C-5) and 29.2 (CH_2_, C-3). All available evidence led to the planar structure **1d** (Figure 2), belonging to germacrane sesquiterpene. 

**Figure 4:**
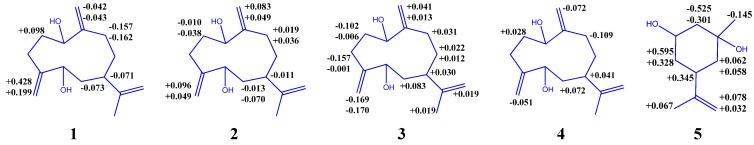
∆*δ*_S-R_ values of MTPA esters of **1**–**5**.

To determine the absolute configuration of C-1 and C-5, the modified Mosher ester procedure was employed [24,25]. Thus, treatment with (*R*)- and (*S*)-MTPA chlorides led to esterification of 1-OH and 5-OH, affording (*S*)- and (*R*)-MTPA derivatives, respectively. The ^1^H-NMR chemical shift differences (∆*δ_S_*_-*R*_) were observed (**1**, Figure 4). The absolute configuration of C-1 and C-5 were consequently determined to be *R* and *S*, respectively. In the NOESY spectrum, *δ*_H_ 2.68 (m, H-7) correlated with *δ*_H_ 4.24 (dd, *J* = 5.2, 10.5 Hz, H-1), suggesting the *R*-configuration of C-7. Thus, **1** was identified to be (1*R*,5*S*,7*R*)-1,5-dihydroxygermacra-4(15),10(14),11(12)-triene. To the best of our knowledge, it is the first time a germacrane sesquiterpene is isolated from *Illicium* plants. 

**Table 1:** ^13^C-NMR spectroscopic data for compounds **1**–**4** (CD_3_OD, 100 MHz).

No.	1 (mult.)	2 (mult.)	3 (mult.)	4 (mult.)
1	77.2 (C)	70.6 (C)	74.1 (C)	75.3 (C)
2	33.9 (CH_2_)	33.5 (CH_2_)	34.4 (CH_2_)	33.5 (CH_2_)
3	29.2 (CH_2_)	26.7 (CH_2_)	27.0 (CH_2_)	25.8 (CH_2_)
4	152.7 (C)	152.2 (C)	153.6 (C)	151.6 (C)
5	72.5 (CH)	74.6 (CH)	76.7 (CH)	77.3 (CH)
6	41.1 (CH_2_)	40.6 (CH_2_)	40.1 (CH_2_)	37.8 (CH_2_)
7	39.7 (CH)	40.8 (CH)	42.1 (CH)	42.2 (CH)
8	30.4 (CH_2_)	30.1 (CH_2_)	31.5 (CH_2_)	33.4 (CH_2_)
9	29.8 (CH_2_)	35.2 (CH_2_)	32.1 (CH_2_)	31.4 (CH_2_)
10	150.6 (C)	152.1 (C)	151.7 (C)	150.6 (C)
11	151.3 (C)	150.7 (C)	150.4 (C)	150.5 (C)
12	110.3 (CH_2_)	110.5 (CH_2_)	110.4 (CH_2_)	110.5 (CH_2_)
13	20.1 (CH_3_)	20.5 (CH_3_)	19.0 (CH_3_)	19.4 (CH_3_)
14	116.9 (CH_2_)	113.8 (CH_2_)	114.3 (CH_2_)	114.4 (CH_2_)
15	112.4 (CH_2_)	111.4 (CH_2_)	112.8 (CH_2_)	114.9 (CH_2_)

**Table 2:** ^1^H-NMR Spectroscopic Data for Compounds **1**–**4** (CD_3_OD, 400 MHz).

No.	1 [mult., *J* (Hz)]	2 [mult., *J* (Hz)]	3 [mult., *J* (Hz)]	4 [mult., *J* (Hz)]
1	4.24 dd (5.2, 10.5)	4.23 dd (4.5, 9.6)	4.11 dd (4.2, 10.4)	4.16 dd (4.1, 9.6)
2	2.10 m; 1.98 m	2.22 m; 1.95 m	2.25 m; 1.82 m	2.09 m; 1.56 m
3	2.29 m	2.38 dd (4.0, 11.4); 2.07 m	2.11 (overlap)	2.32 dd (4.0, 14.2); 2.07 m
5	4.36 dd (3.9, 7.0)	4.31 dd (3.5, 5.9)	3.88 t (7.8)	3.92 dd (3.9, 11.4)
6	1.83 m	1.91 ddd (2.3, 6.4, 14.2); 1.69 (overlap)	1.67 (overlap)	1.85 ddd (2.7, 11.3, 14.0); 1.59 m
7	2.68 m	2.42 m	2.11 (overlap)	2.17 m
8	1.68 m	1.74 m; 1.53 m	1.61 m	1.96 m; 1.64 m
9	2.45 m; 2.04 m	2.20 m; 2.13 dd (3.0, 11.2)	2.14 (overlap)	2.42 m; 2.05 m
12	4.77 s	4.73 s; 4.69 s	4.66 br s	4.69 s; 4.68 s
13	1.76 s	1.70 s	1.67 s	1.69 s
14	5.16 s; 4.95 s	5.19 s; 5.03 s	5.24 s; 5.02 s	5.18 s; 5.02 s
15	5.13 s; 5.02 s	5.05 s; 4.98 s	5.12 s; 5.05 s	5.02 br s

Compounds **2**, **3**, and **4** were stereoisomers of **1**. By using the same strategies as in the structural elucidation of **1**, compounds **2**, **3**, and **4** were identified to be (1*S*,5*S*,7*R*)-1,5-dihydroxygermacra-4(15),10(14),11(12)-triene, (1*S*,5*R*,7*R*)-1,5-dihydroxygermacra-4(15),10(14),11(12)-triene, and (1*R*,5*R*,7*R*)-1,5-dihydroxygermacra-4(15),10(14),11(12)-triene (Figure 1 and Figure 4), respectively. In the NOESY spectrum of **2**, *δ*_H_ 1.70 (s, H-13) correlated with *δ*_H_ 4.31 (dd, *J* = 3.5, 5.9, H-5), indicating the *α*-orientation of H-7. For compound **3**, due to the unresolved overlap of the H-7 signal in CD_3_OD, NOESY spectrum was further acquired in CDCl_3_ for the assignment of relative configuration of H-7. The *α*-orientation of H-7 was suggested by the NOESY correlation between H-7 and 5*α*-H. While the *α*-orientation of H-7 in **4** was suggested by the NOESY correlation from *δ*_H_ 2.17 (m, H-7) to both *δ*_H_ 4.16 (dd, *J* = 4.1, 9.6, H-1) and *δ*_H_ 3.92 (dd, *J* = 3.9, 11.4, H-5). Compound **4** is a known structure, previously reported from *Gonospermum elegans* but with undetermined absolute configuration [26]. 

Compound **5** was obtained as a white amorphous powder. A molecular formula of C_10_H_18_O_2_ was determined based on the HRESIMS result at *m/z* 193.1202 [M + Na]^+^ (calcd. 193.1199), indicating two degrees of unsaturation. The ^1^H-, ^13^C- and DEPT-NMR spectra (Table 3) revealed the presence of two methyls, four methylenes (including one olefinic ones), two methines (including one oxygenated group), and two quaternary carbons (including an olefinic one). All proton signals were assignable based on the gHSQC experiment (Table 3). The ^1^H-^1^H COSY spectrum displayed correlations between *δ*_H_ 1.72 (H-2) and *δ*_H_ 3.60 (d, *J* = 3.16 Hz, H-3), between *δ*_H_ 3.60 (H-3) and *δ*_H_ 1.91 (ddd, *J* = 2.8, 11.6, 14.0 Hz, H-4a) and *δ*_H_ 1.63 (dt, *J* = 3.4, 13.8 Hz, H-4b), between *δ*_H_ 1.91 (H-4a) and *δ*_H_ 2.23 (m, H-5), between *δ*_H_ 2.23 (H-5) and *δ*_H_ 1.53 (H-6), leading to the connections of between C_2_-C_3_-C_4_-C_5_-C_6_ (Figure 1 and Figure 3). The HMBC correlations between *δ*_H_ 1.69 (s, 10-H) and *δ*_C_ 37.4 (C-5), *δ*_C_ 109.0 (C-9), and *δ*_C_ 149.2 (C-8), as well as correlations between *δ*_H_ 4.70 (br s, H-9) and *δ*_C_ 21.1 (C-10), *δ*_C_ 37.4 (C-5), and *δ*_C_ 149.2 (C-8), suggested that C-9 and C-10 were connected to C-5 via C-8 (Figure 1 and Figure 3). The remaining part of the structure was established based on the following long-range correlations: *δ*_H_ 1.53 (H-6)/*δ*_C_ 71.4 (C-1); *δ*_H_ 1.23 (s, H-7)/*δ*_C_ 71.4 (C-1) and *δ*_C_ 33.7 (C-2). Two hydroxyl groups were assigned to C-1 and C-3, respectively. In the NOESY spectrum, *δ*_H_ 2.23 (m, H-5) displayed correlations with *δ*_H_ 3.60 (d, *J* = 3.2 Hz, H-3), and *δ*_H_ 1.23 (s, H-7), indicating the relative stereochemistry of 1*β*,3*β*-dihydroxy-(5*α*H)-*m*-menth-8-ene. To determine the absolute stereochemistry, modified Mosher’s ester procedure was carried out. Due to the steric hindrance on C-1, only 3-OH was esterified by (*R*)- and (*S*)-MTPA chlorides into (*S*)- and (*R*)-MTPA derivatives. The absolute configuration of C-3 was finally deduced to be *R* based on the proton shift difference between (*S*)- and (*R*)-MTPA derivatives (**5**, Figure 4), indicating a 3*α*-H. The 1*S*- and 5*S*-configuration were then assignable based on the NOESY results mentioned above. Consequently, **5** was determined to be (1*S*,3*R*,5*S*)-1,3-dihydroxy-*m*-menth-8-ene.

**Table 3:** ^13^C- and ^1^H-NMR spectroscopic data for compound **5** (CDCl_3_) *.

No.	*δ*_C_ (mult.)	*δ*_H_ [mult., *J* (Hz)]
1	71.4 (C)	-
2	33.7 (CH_2_)	1.72 (partial overlap); 1.49 (partial overlap)
3	73.8 (CH)	3.60 d (3.2)
4	34.0 (CH_2_)	1.91 ddd (2.8, 11.6, 14.0); 1.63 dt (3.4, 13.8)
5	37.4 (CH)	2.23 m
6	26.1 (CH_2_)	1.53 (partial overlap)
7	26.5 (CH_3_)	1.23 s
8	149.2 (C)	-
9	109.0 (CH_2_)	4.70 br s
10	21.1 (CH_3_)	1.69 s

* 100 MHz for ^13^C and 400 MHz for ^1^H, respectively.

Compounds **6**, **7**, and **8** were identified to be 3-hydroxyocta-1,5*E*-dien-7-one [27], 2-(4-methylphenyl)-1,2-propanediol [28], and *trans*-3,4,5-trimethoxycinnamic alcohol [29], respectively, based on the interpretation of their NMR spectroscopic data and comparison with reported data. **6**, **7**, and **8** were isolated from *Illicium* plants for the first time.

Compounds **3** and **4** exhibited proliferative activity in SH-SY5Y cells at concentrations of 0.49 µM–125 µM. Compounds **3** (at 62.5 µM) and **4** (at 15.6 µM) could promote proliferation by 36.2% and 45.8% after 48-h incubation, respectively. Compounds **5**–**8** were inactive; **6** and **8** displayed cytotoxicity at the concentrations above 50 µM. Due to the scarcity of **1** and **2**, they were not tested for the bioactivity. Efforts to obtained additional crops of these compounds are in progress.

## 3. Experimental

### 3.1. General

The FT-IR spectra (KBr) were recorded using a Thermo FT-IR Nicolet 5700. Optical rotations at sodium D line were measured with a Perkin-Elmer 241 digital polarimeter using quartz cell with a path length of 100 mm at room temperature. Concentrations (c) are given in g/100 mL. Nuclear magnetic resonance (NMR) spectra were recorded on a Bruker DPX-400 or AVANCE-400 spectrometer running at 400 MHz for ^1^H and 100 MHz for ^13^C, respectively. All chemical shifts were quoted on the *δ* scale in ppm using residual solvent as the internal standard (CDCl_3_: 7.24 ppm for ^1^H-NMR, 77.0 ppm for ^13^C-NMR; CD_3_OD: 3.30 ppm for ^1^H-NMR, 49.0 ppm for ^13^C-NMR; C_5_D_5_N: 8.71 ppm, 7.55 ppm, 7.19 ppm for ^1^H-NMR, 149.9 ppm, 135.5 ppm, 123.5 ppm for ^13^C-NMR). Coupling constants (*J*) are reported in Hz. The following abbreviations are used to indicate the multiplicity: s = singlet, d = doublet, t = triple, dd = double doublet, dt = double triplet, br = broad. Thin layer chromatography (TLC) was carried out using Merck aluminium backed sheets coated with 60F254 silica gel or 60F254 RP-silica gel. Visualization of the plates was achieved by using a UV lamp (λ_max_ = 254 nm), and spraying a mixture of 2% *p*-hydroxybenzaldehyde methanolic solution and 5% sulphuric acid ethanolic solution (10:1, *v/v*) followed by heating. Open column chromatography was carried out on columns packed with silica gel, RP silica gel (C_18_) (Macherey-Nagel GmbH & Co. KG, Düren, Germany), MCI gel CPH 20 (Supelco, Sigma-Aldrich, Bellefonte, PA, USA), and Sephadex LH-20 (GE Healthcare Bio-Sciences AB, Uppsala, Sweden). For HPLC purification, a C_18_ semi-preparative HPLC column (Phenomenex C_18_ column, 250 × 10 mm, 5 μm) and a Shimadzu UFLC system were used; the UV detection wavelength and flow rate were set at UV_210nm_ and 4 mL/min, respectively. A Shimadzu UFLC XR system coupled with a LCMS-2020 liquid chromatography mass spectrometer was used for sample analysis. All solvents used were analytical or HPLC grade. HRESIMS were measured on a Shimadzu LCMS-IT-TOF Mass Spectrometry. 

### 3.2. Plant Material

The pericarps of *Illicium lanceolatum* A. C. Smith. were collected from An-hui Province, China, in October 2011, and were identified by one of the authors (Jin-Ao Duan). A voucher specimen was deposited in the Department of Medicinal Chemistry and Pharmacognosy (UICMCP001-Ilan-P), College of Pharmacy, University of Illinois at Chicago, Chicago, IL, USA.

### 3.3. Extraction and Isolation

The dried pericarps of *Illicium lanceolatum* (1 kg) were powdered and extracted by percolation with methanol (MeOH, 15 L), yielding 300 g of extract. The extract was applied to a silica gel flash column eluted with mixtures of petroleum ether (PE)-ethyl acetate (EA) (100:0 → 50:50), followed by dichloromethane (DCM)-MeOH (90:10, 85:15, and 0:100), to yield 40 fractions (A_1_–A_40_). The fractions after A_35_ (A_35_: elute of DCM-MeOH, 85:15) contained plenty of shikimic acid. Fraction A_17_ was further fractionated into 23 fractions (B_1_–B_23_) on a silica gel column eluted with mixtures of PE-EA (70:30 and 60:40). B_11_ was subjected to RP-18 chromatography eluted with aqueous MeOH (20% and 100%) to yield 15 fractions (C_1_–C_15_). Compound **6** (6.1 mg) was purified from fractions of C_4_ and C_5_ by Sephadex LH-20 chromatography (eluted with 30% aqueous MeOH). Fractions of C_8_–C_14_ were combined and subjected to Sephadex LH-20 chromatography (eluted with 30% aqueous MeOH) to obtain a mixture (>100 mg) of compounds **5** and **7**. An aliquot of the mixture was purified by semi-preparative HPLC using 30% aqueous acetonitrile (AcCN) as mobile phase to yield compounds **5** (>50 mg), and **7** (5 mg). Fractions A_20_–A_22_ were further fractionated into 25 subfractions (D_1_–D_25_) on a MCI column eluted with aqueous methanol (10% → 100%). D_14_ and D_15_ were subjected to Sephadex LH-20 chromatography (eluted by MeOH) to yield 20 fractions (E_1_–E_20_). Compound **8** (18 mg) was purified from E_13_–E_15_ by semi-preparative HPLC separation (35% aqueous AcCN). E_7_ and E_8_ were further subjected to a silica gel column eluted with mixtures of PE-EA (80:0 → 65:35) to yield 32 fractions (F_1_–F_32_). There were three main components in fractions of F_13_–F_16_. They were purified by semi-preparative HPLC chromatography (35% aqueous MeOH) to yield compounds **1** (2.5 mg), **2** (2.2 mg). Compounds **3** (15 mg) and **4** (50 mg) were purified from F_17_–F_26_ by semi-preparative HPLC chromatography (35% aqueous MeOH).

### 3.4. Spectral Data

*(1R,5S,7R)-1,5-Dihydroxygermacra-4(15),10(14),11(12)-triene* (**1**). White amorphous powder. IR (cm^−1^): 3377, 2989, 1703, 1576, 1405, 1081. 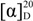 −3° (*c* 0.12, MeOH). ^13^C- and ^1^H-NMR spectroscopic data (CD_3_OD): see Table 1 and Table 2, respectively. HR-ESI-MS *m/z* 219.1733 [M−H_2_O+H]^+^ (calcd. for C_15_H_23_O, 219.1743), *m/z* 201.1639 [M-−2H_2_O+H]^+^.

*(1S,5S,7R)-1,5-Dihydroxygermacra-4(15),10(14),11(12)-triene* (**2**). Colorless oil. IR (cm^−1^): 3359, 2928, 1641, 1439, 1015, 895. 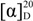 −7° (*c* 0.14, MeOH). ^13^C- and ^1^H-NMR spectroscopic data (CD_3_OD): see Table 1 and Table 2, respectively. HR-ESI-MS *m/z* 219.1735 [M−H_2_O+H]^+^ (calcd. for C_15_H_23_O, 219.1743), 201.1630 [M−2H_2_O+H]^+^.

*(1S,5R,7R)-1,5-Dihydroxygermacra-4(15),10(14),11(12)-triene* (**3**). White amorphous powder. IR (cm^−1^): 3260, 2928, 1644, 1434, 1020, 905. 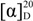 −4° (*c* 0.17, MeOH). ^13^C- and ^1^H-NMR spectroscopic data (CD_3_OD): see Table 1 and Table 2, respectively. ^1^H spectroscopic data (CDCl_3_, 400 Hz): *δ*_H_ 5.24 (1H, s, H-14a), 5.03 (1H, s, H-14b); 5.16 (1H, s, H-15a), 5.06 (1H, s, H-15b); 4.65 (1H, s, H-12a), 4.64 (1H, s, H-12b); 4.16 (1H, dd, *J* = 4.3, 10.2 Hz, H-1); 3.95 (1H, dd, *J* = 5.0, 10.7 Hz, H-5); 2.27 (1H, m, H-2a), 1.83 (1H, m, H-2b); 2.13 (4H, overlapped, H-3 and H-9); 2.06 (1H, t, *J* = 5.7 Hz, H-7); 1.73 (1H, t, *J* = 5.2 Hz, H-6a), 1.59 (1H, partially overlapped, H-6b); 1.58 (2H, partially overlapped, H-8a; 1.65 (3H, s, H-13). HR-ESI-MS *m/z* 219.1734 [M−H_2_O+H]^+^ (calcd. for C_15_H_23_O, 219.1743), 201.1642 [M−2H_2_O+H]^+^, 237.1820 [M+H]^+^. 

*(1R,5R,7R)-1,5-Dihydroxygermacra-4(15),10(14),11(12)-triene* (**4**). Colorless oil. IR (cm^−1^): 3368, 2927, 1642, 1449, 1012, 893. 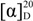 +11° (*c* 0.16, MeOH). ^13^C- and ^1^H-NMR spectroscopic data (CD_3_OD): see Table 1 and Table 2, respectively. HR-ESI-MS *m/z* 219.1740 [M−H_2_O+H]^+^ (calcd. for C_15_H_23_O, 219.1743), 201.1650 [M−2H_2_O+H]^+^.

*(1S,3R,5S)-1,3-Dihydroxy-m-menth-8-ene* (**5**). White amorphous powder. ^13^C- and ^1^H-NMR spectroscopic data (CDCl_3_): see Table 3. HR-ESI-MS* m/z* 193.1202 [M+Na]^+^ (calcd. for C_10_H_18_O_2_Na, 193.1199).

### 3.5. Preparation of the (R)- and (S)-MTPA Ester Derivatives of **1**–**5**

In these experiments, (*R*)- and (*S*)-MTPA chloride was used to react with each compound to yield its (*S*)- and (*R*)-MTPA derivatives, respectively [25]. Two aliquots of compound (0.5–1.0 mg each) were transferred into two NMR tubes and dried overnight in a desiccator with P_2_O_5_ inside. After successive addition of 6 μL of (*R*)- or (*S*)-MTPA chloride and 600 μL of pyridine-*d_5_*, the NMR tubes were sealed immediately, and shaken vigorously. The tubes were then kept in desiccator overnight until the reaction was complete [30]. The ^1^H-NMR spectra of the final (*R*)- and (*S*)-MTPA derivatives were recorded, and the chemical shifts were assigned based on the ^1^H-^1^H COSY NMR experiments. In case that signals could not be unambiguously assigned, gHSQC and gHMBC experiments were carried out. The ∆*δ_S_*_-*R*_ values were calculated [24,25].

### 3.6. In Vitro Assay on SH-SY5Y

The SH-SY5Y cells were maintained in the Opti-MEM with 10% FBS, 100 U/mL penicillin and 100 µg/mL streptomycin. The cells (5 × 10^4^ or 1 × 10^5^/well in 1 mL growth medium) were incubated with various concentrations of compounds in 24-well culture plates. After 48-h incubation, 20 µL MTT (3-(4,5-dimethylthiazol-2yl)-2.5-diphenyltetrazolium bromide, 5 mg/mL in PBS) were added to the each well. The supernatant were removed after further 4 h incubation. The formazan in each well were dissolved in 300 µL isopropanol with 4 mM HCl and 0.1% Nondet P-40. The absorbance was read at 590 nm with a reference filter of 620 nm by using microplate reader (Infinite M200 Pro, Tecan, San Jose, CA, USA). The cells without treatment were as vehicle control. The cells were treated by corresponding concentration of DMSO as control. The percentage of growth promotion was calculated using the following formula: % cell promotion = 100 × (OD_590nm test compound_ − OD_590nm control_)/OD_590nm control_. Results were expressed as the mean of at least three independent experiments.

## 4. Conclusions

Three new germacrane sesquiterpenes **1**–**3**, a new *m*-menthane monoterpene **5**, together with a known germacrane sesquiterpene **4**, and three other known compounds **6**–**8** have been identified from *I. lanceolatum*. All the compounds were isolated from *Illicium* plants for the first time. Absolute stereochemistry for germacranes and *m*-menthane were established. Compounds **3** and **4** exhibited proliferative activity on the SH-SY5Y cell lines, indicating potential neurotrophic activity. Further biological evaluation on primary cultures of fetal rat cortical neurons is under planning.

## Supplementary Files

Supplementary materials can be accessed at: http://www.mdpi.com/1420-3049/19/4/4326/s1.
