# Supplementary material for: Germacranes and m-Menthane from Illicium lanceolatum
Source: Molecules. 2014 Apr 4;19(4):4326–37. doi: 10.3390/molecules19044326 (PMC6271484; doi:10.3390/molecules19044326)

Supplementary Figures

For Compound **1**

Figure S1. ^{1}H-NMR spectrum of Compound **1** in CD_3OD.
Figure S2. ^{13}C-NMR spectrum of Compound **1** in CD_3OD.
Figure S3. ^{1}H-^{1}H COSY spectrum of Compound **1** in CD_3OD.
Figure S4. gHSQC spectrum of Compound **1** in CD_3OD.
Figure S5. gHMBC spectrum of Compound **1** in CD_3OD.
Figure S6. NOESY spectrum of Compound **1** in CD_3OD.
Figure S7. HRESIMS spectrum of Compound **1**.
Figure S8. Absolute configuration determination for C-1 and C-5 in **1** by modified Mosher's ester method.

For Compound **2**

Figure S9. ^{1}H-NMR spectrum of Compound **2** in CD_3OD.
Figure S10. ^{13}C-NMR spectrum of Compound **2** in CD_3OD.
Figure S11. ^{1}H-^{1}H COSY spectrum of Compound **2** in CD_3OD.
Figure S12. gHSQC spectrum of Compound **2** in CD_3OD.
Figure S13. gHMBC spectrum of Compound **2** in CD_3OD.
Figure S14. NOESY spectrum of Compound **2** in CD_3OD.
Figure S15. HRESIMS spectrum of Compound **2**.

For Compound **3**

Figure S16. ^{1}H-NMR spectrum of Compound **3** in CD_3OD.
Figure S17. ^{1}H-NMR spectrum of Compound **3** in $CDCl_3$.
Figure S18. ^{13}C-NMR spectrum of Compound **3** in CD_3OD.
Figure S19. ^{1}H-^{1}H COSY spectrum of Compound **3** in CD_3OD.
Figure S20. gHSQC spectrum of Compound **3** in CD_3OD.
Figure S21. gHMBC spectrum of Compound **3** in CD_3OD.
Figure S22. NOESY spectrum of Compound **3** in CD_3OD.
Figure S23. NOESY spectrum of Compound **3** in $CDCl_3$.
Figure S24. HRESIMS spectrum of Compound **3**.

For Compound **4**

Figure S25. ^{1}H-NMR spectrum of Compound **4** in CD_3OD.
Figure S26. ^{13}C-NMR spectrum of Compound **4** in CD_3OD.
Figure S27. ^{1}H-^{1}H COSY spectrum of Compound **4** in CD_3OD.
Figure S28. gHSQC spectrum of Compound **4** in CD_3OD.
Figure S29. gHMBC spectrum of Compound **4** in CD_3OD.
Figure S30. NOESY spectrum of Compound **4** in CD_3OD.
Figure S31. HRESIMS spectrum of Compound **4**.

For Compound **5**

Figure S32. ^{1}H-NMR spectrum of Compound **5** in $CDCl_3$.
Figure S33. ^{13}C-NMR spectrum of Compound **5** in $CDCl_3$.
Figure S34. ^{1}H-^{1}H COSY spectrum of Compound **5** in $CDCl_3$.
Figure S35. gHSQC spectrum of Compound **5** in $CDCl_3$.
Figure S36. gHMBC spectrum of Compound **5** in $CDCl_3$.
Figure S37. NOESY spectrum of Compound **5** in $CDCl_3$.
Figure S38. HRESIMS spectrum of Compound **5**.

Figure S1. ^{1}H-NMR spectrum of Compound **1** in CD_3OD.

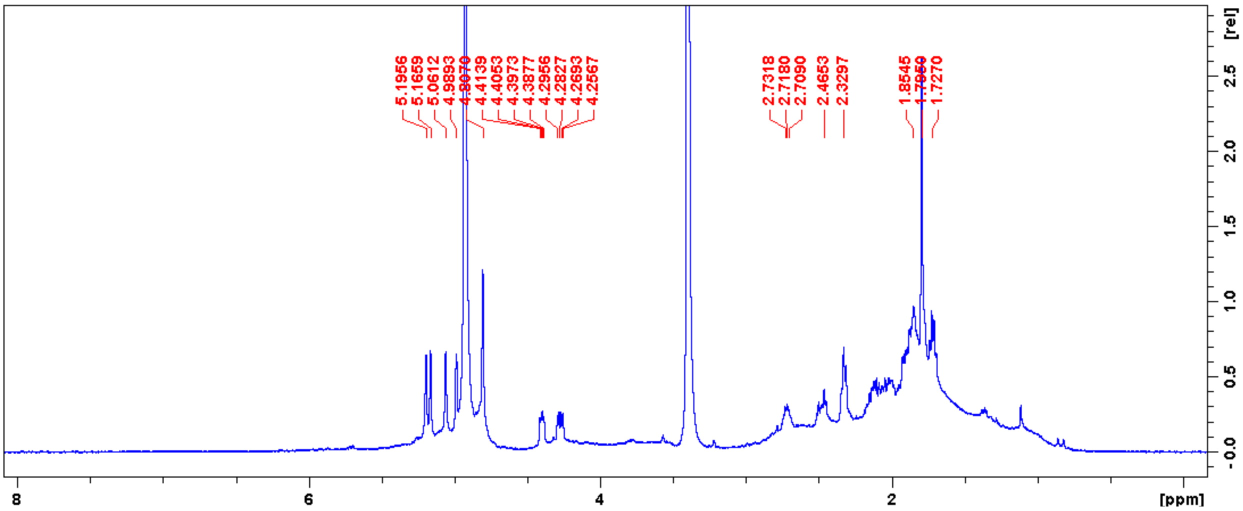

Figure S2. ^{13}C-NMR spectrum of Compound **1** in CD_3OD.

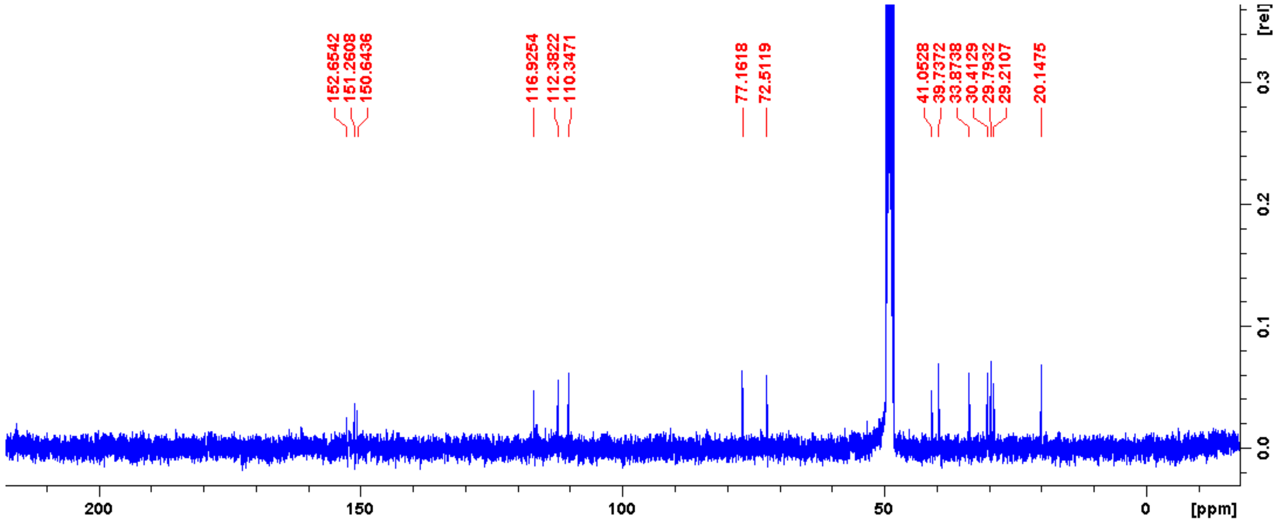

Figure S3. ^{1}H-^{1}H COSY spectrum of Compound **1** in CD_3OD.

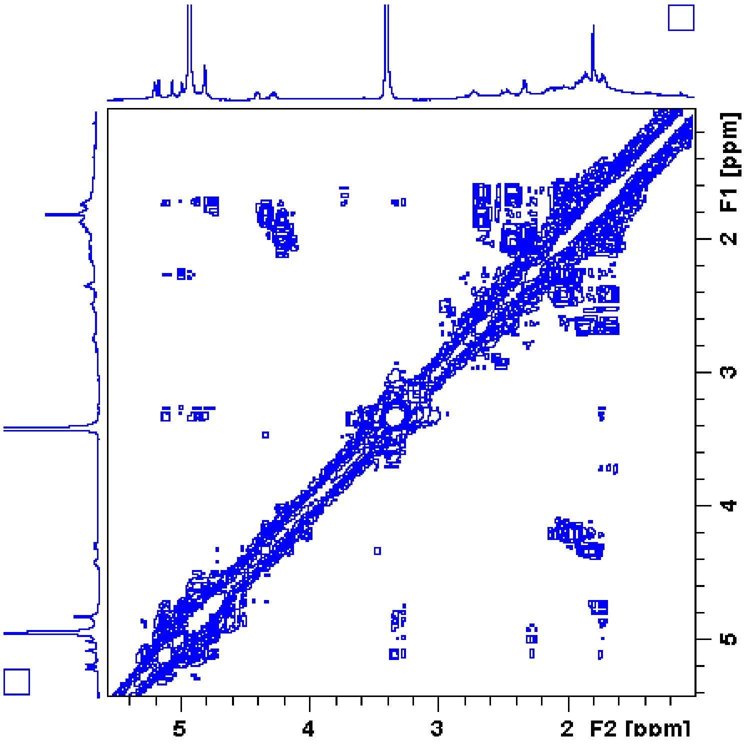

Figure S4. gHSQC spectrum of Compound **1** in CD_3OD.

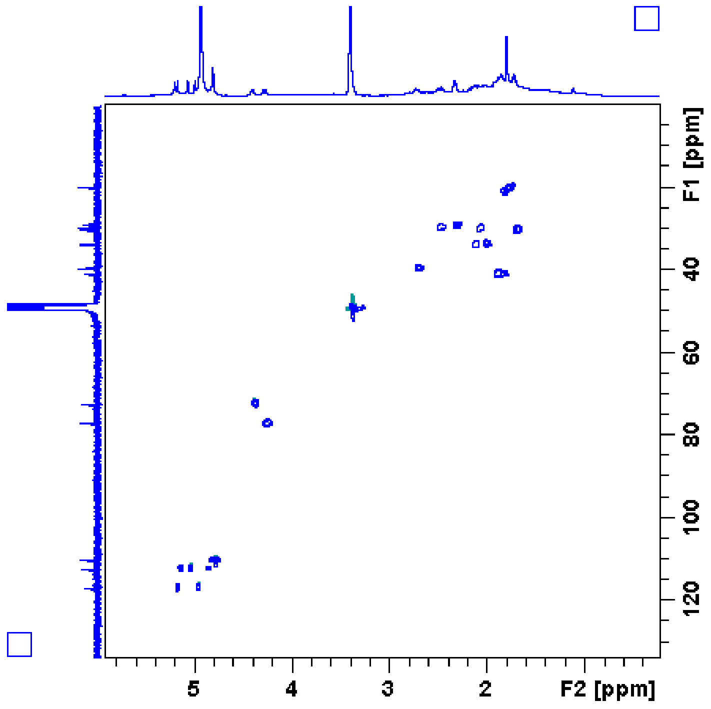

Figure S5. gHMBC spectrum of Compound **1** in CD_3OD.

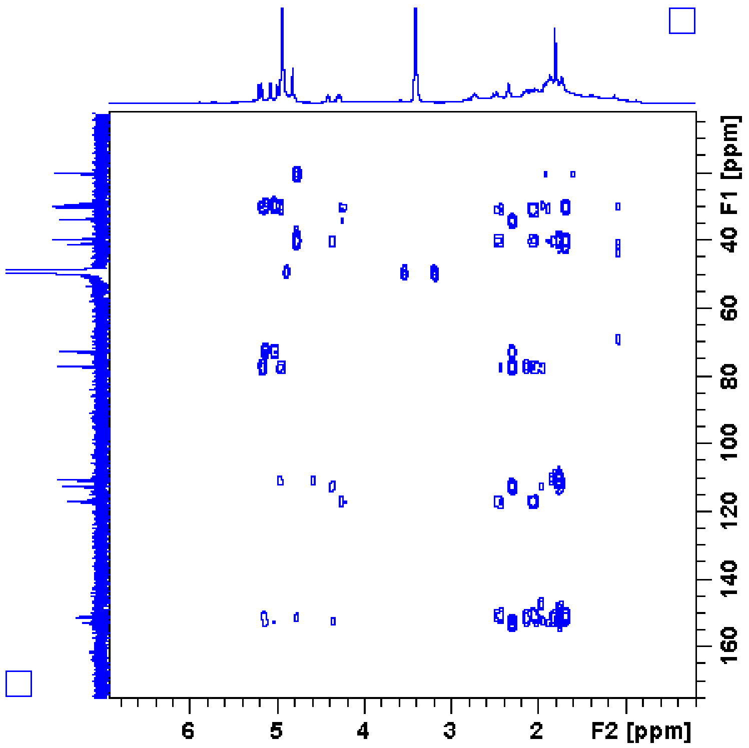

Figure S6. NOESY spectrum of Compound **1** in CD_3OD.

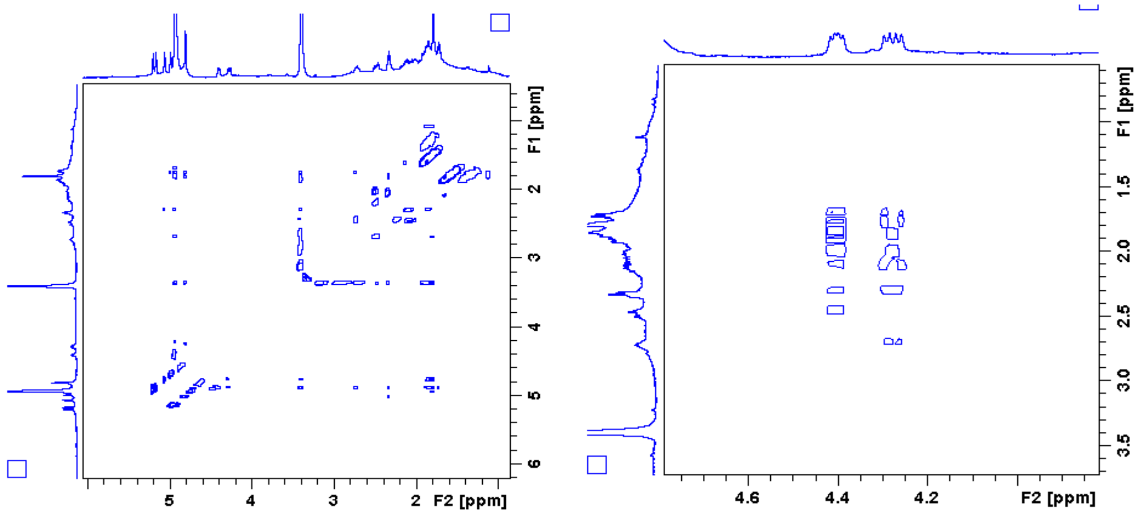

Figure S7. HRESIMS spectrum of Compound **1**.

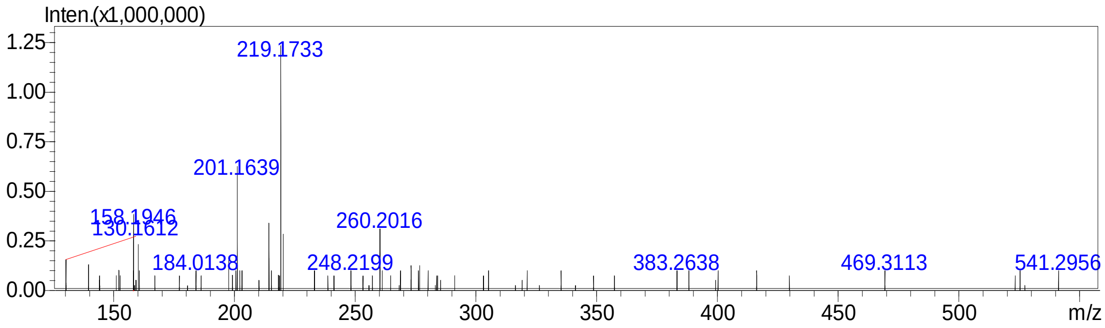

Figure S8. Absolute configuration determination for C-1 and C-5 in **1** by modified Mosher's ester method.

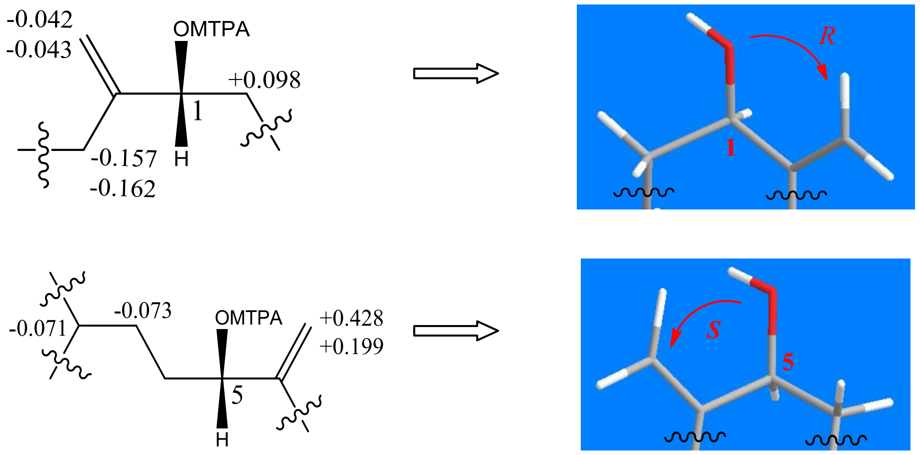

Figure S9. ^{1}H-NMR spectrum of Compound **2** in CD_3OD.

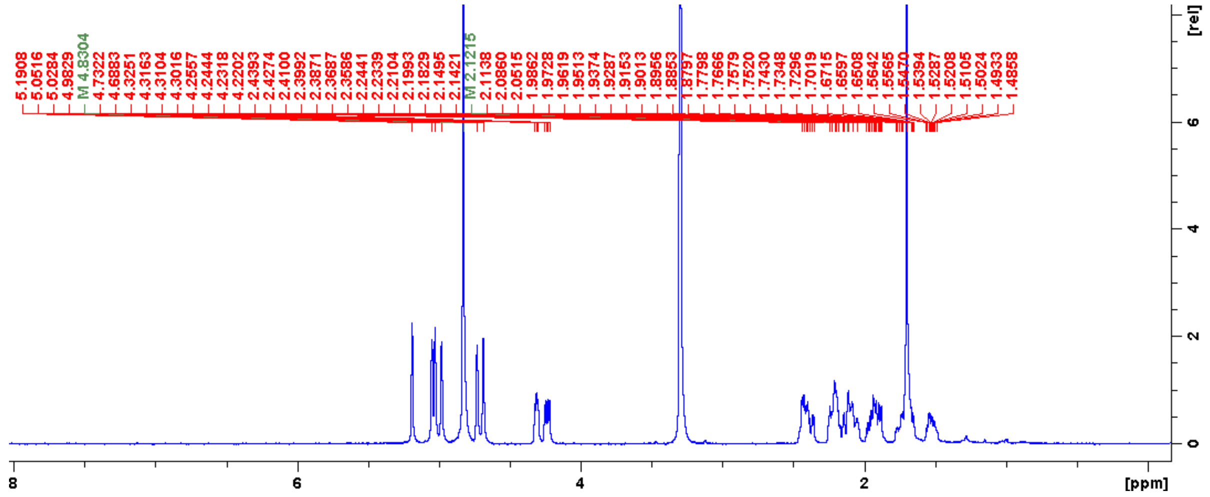

Figure S10. ^{13}C-NMR spectrum of Compound **2** in CD_3OD.

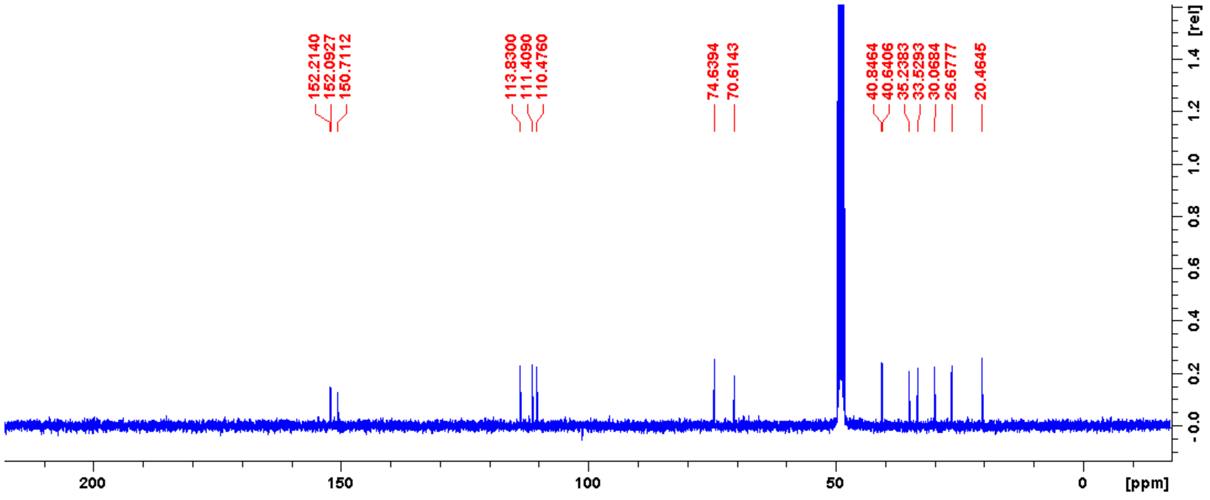

Figure S11. ¹H-¹H COSY spectrum of Compound **2** in CD_3OD.

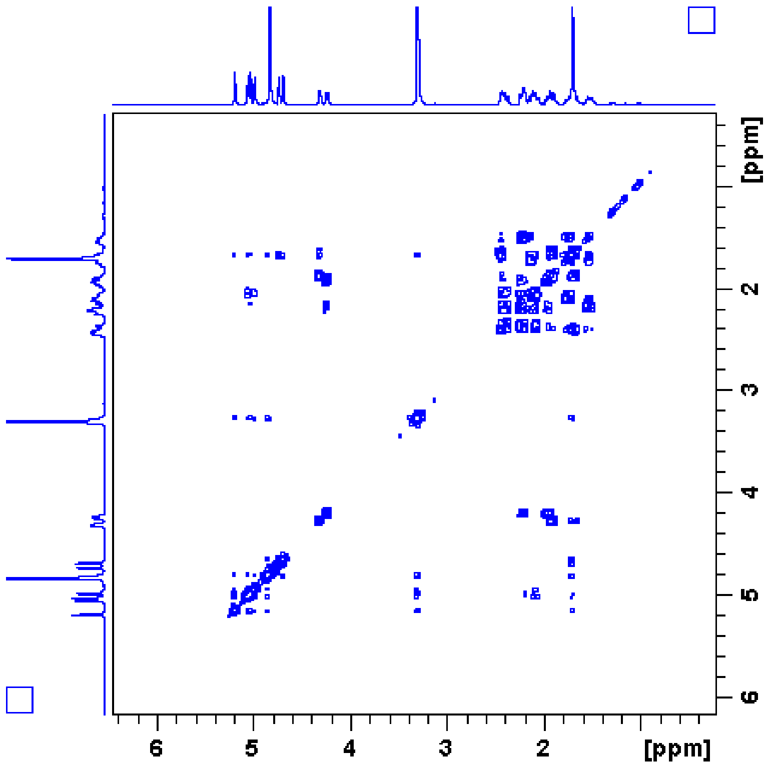

Figure S12. gHSQC spectrum of Compound **2** in CD_3OD.

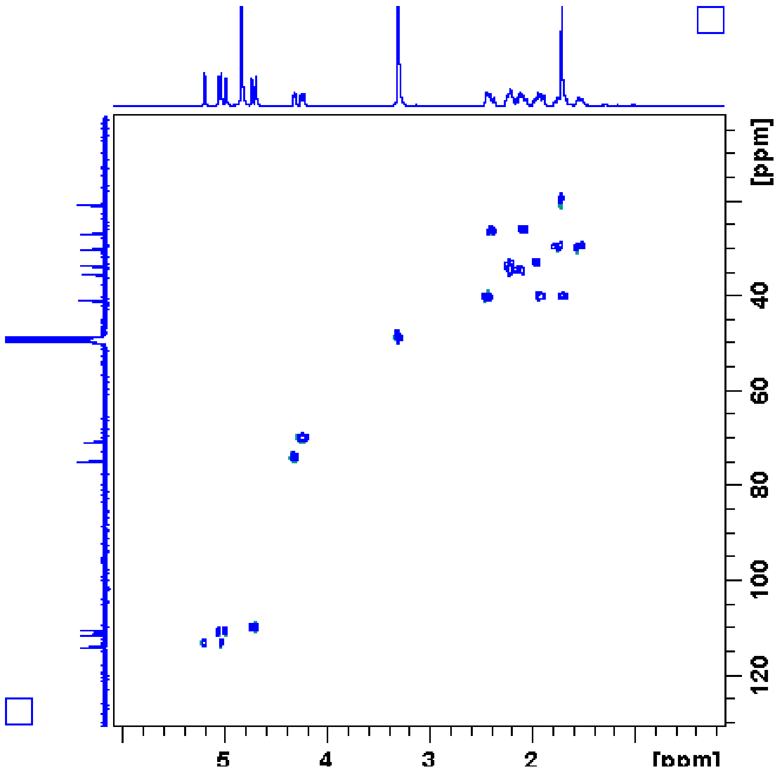

Figure S13. gHMBC spectrum of Compound **2** in CD_3OD.

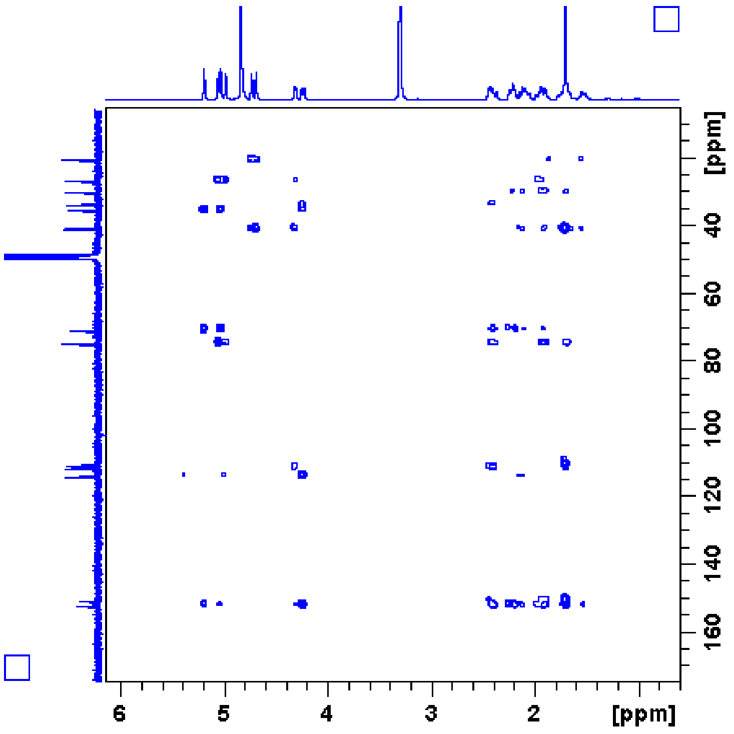

Figure S14. NOESY spectrum of Compound **2** in CD_3OD.

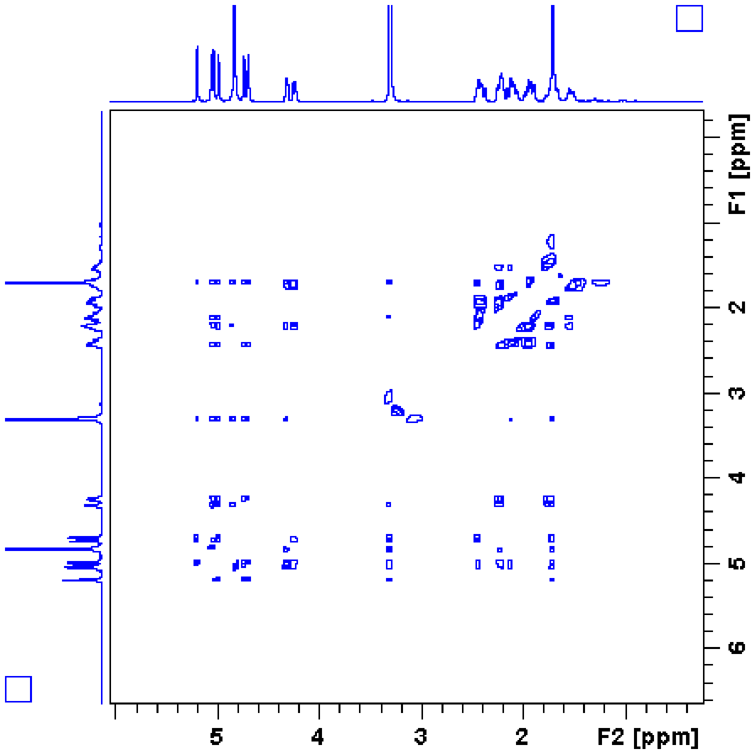

Figure S15. HRESIMS spectrum of Compound **2**.

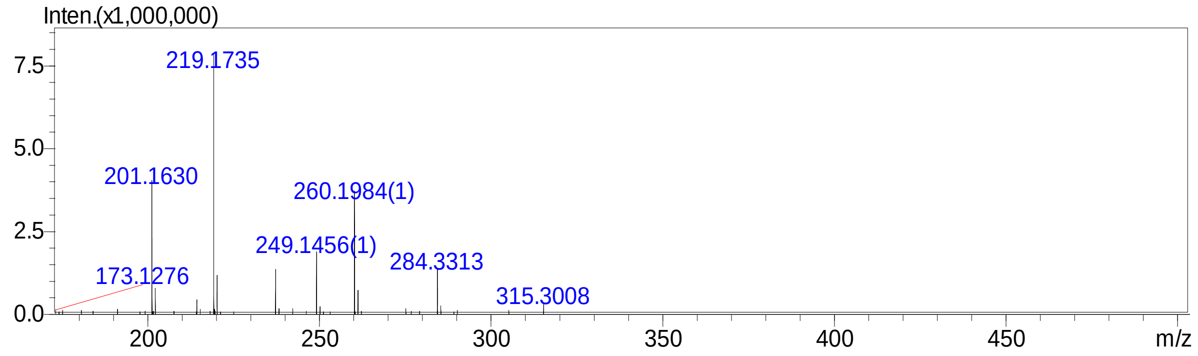

Figure S16. ^{1}H-NMR spectrum of Compound **3** in CD_3OD.

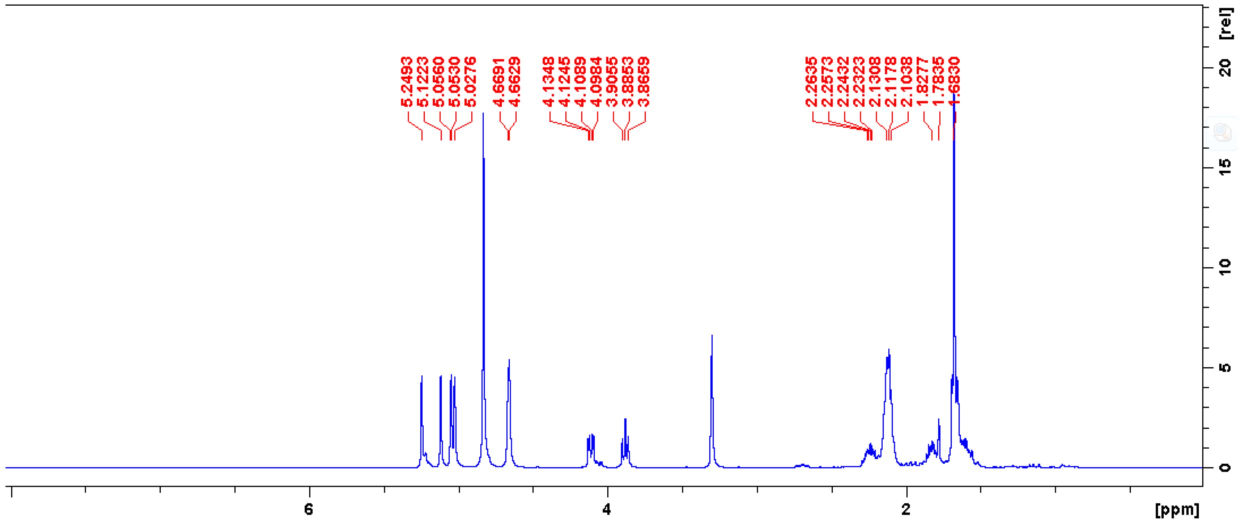

Figure S17. ^{1}H-NMR spectrum of Compound **3** in $CDCl_3$.

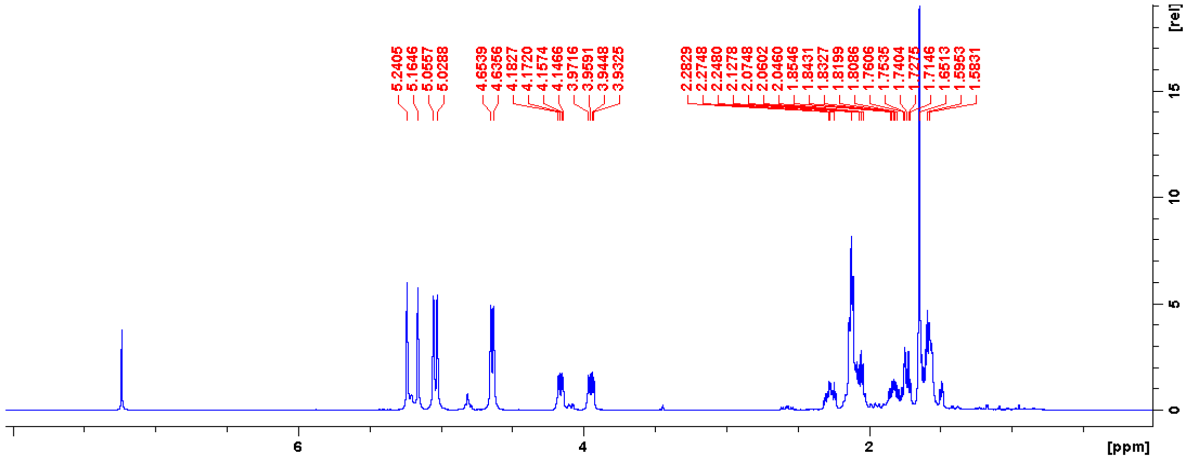

Figure S18. ^{13}C-NMR spectrum of Compound **3** in CD_3OD.

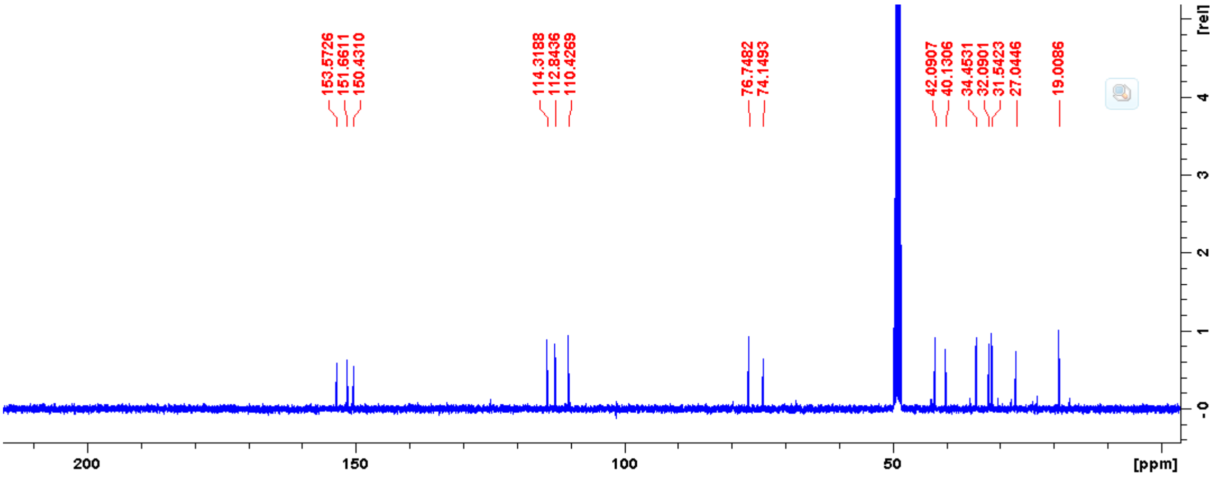

Figure S19. ^{1}H-^{1}H COSY spectrum of Compound **3** in CD_3OD.

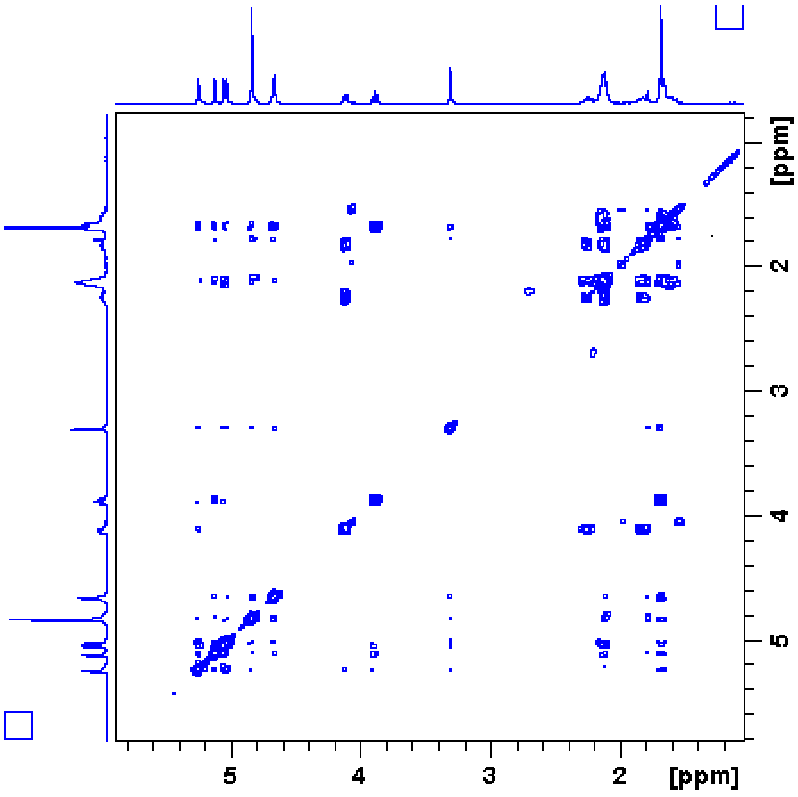

Figure S20. gHSQC spectrum of Compound **3** in CD_3OD.

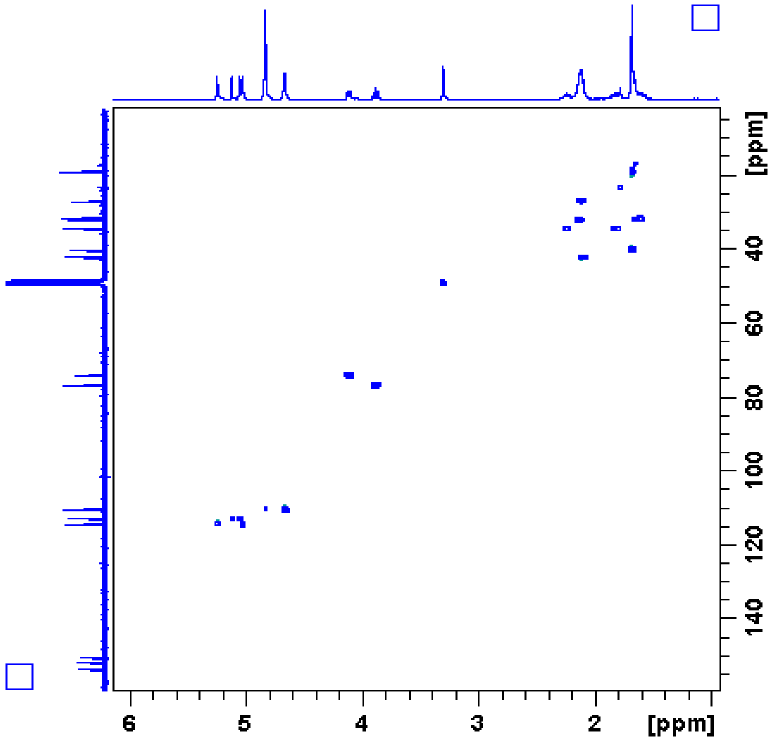

Figure S21. gHMBC spectrum of Compound **3** in CD_3OD.

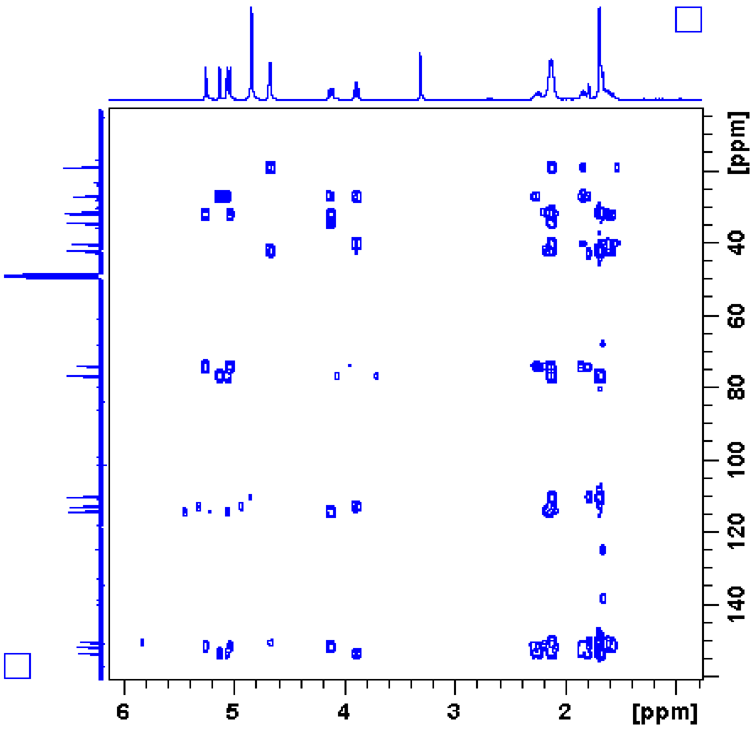

Figure S22. NOESY spectrum of Compound **3** in CD_3OD.

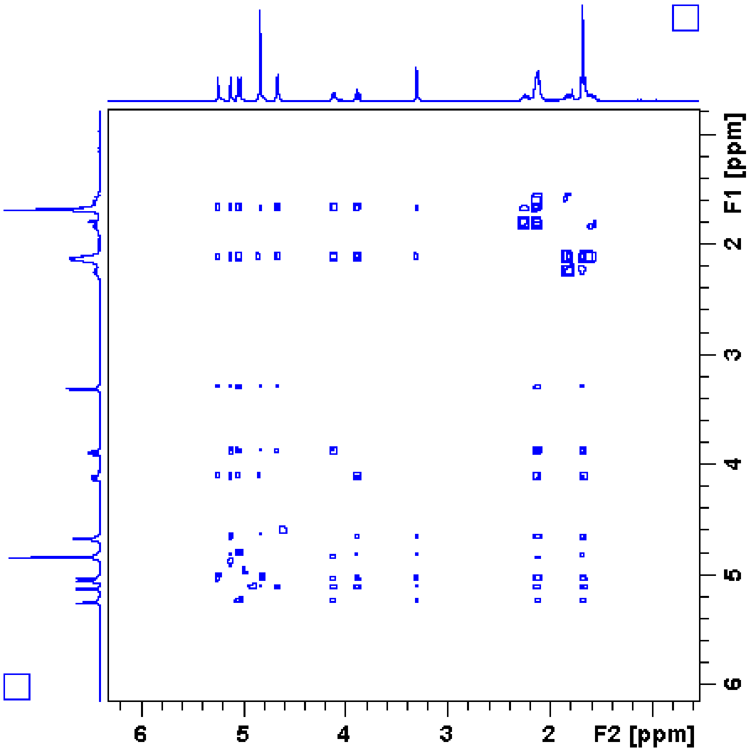

Figure S23. NOESY spectrum of Compound **3** in $CDCl_3$.

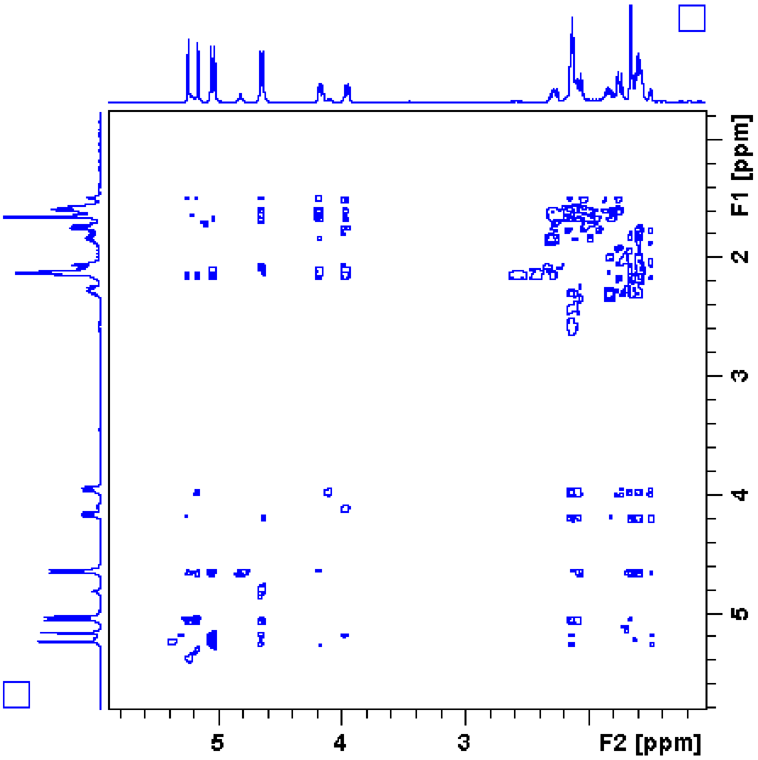

Figure S24. HRESIMS spectrum of Compound **3**.

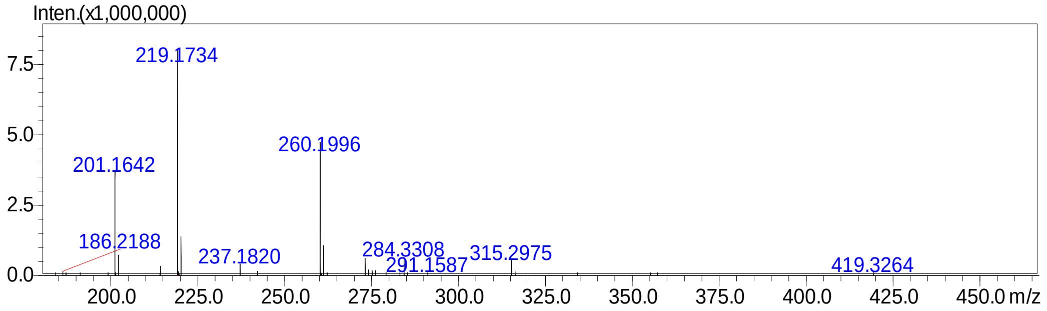

Figure S25. ^{1}H-NMR spectrum of Compound **4** in CD_3OD.

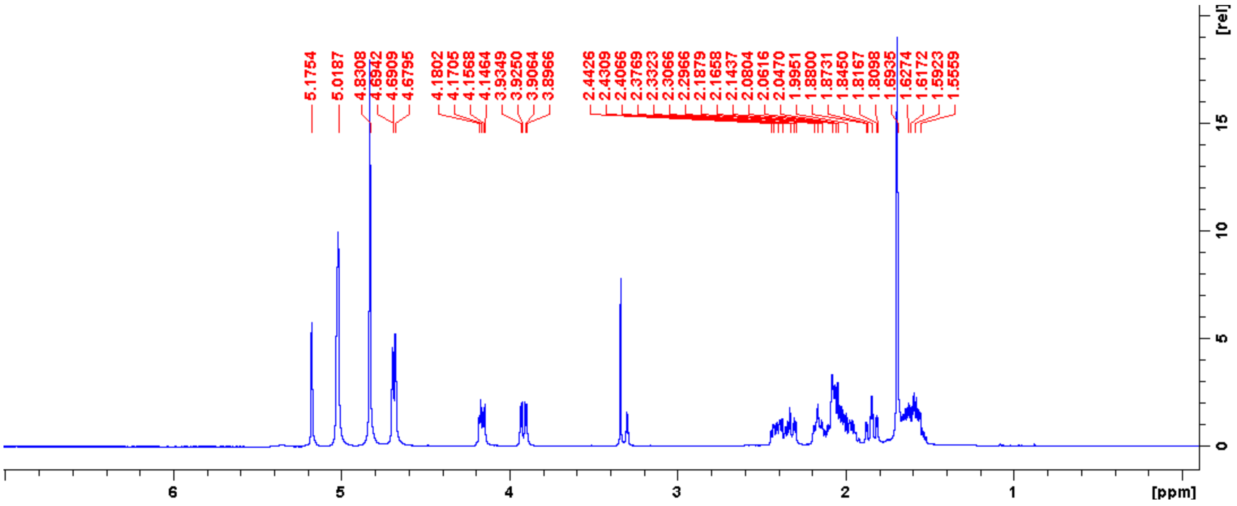

Figure S26. ^{13}C-NMR spectrum of Compound **4** in CD_3OD.

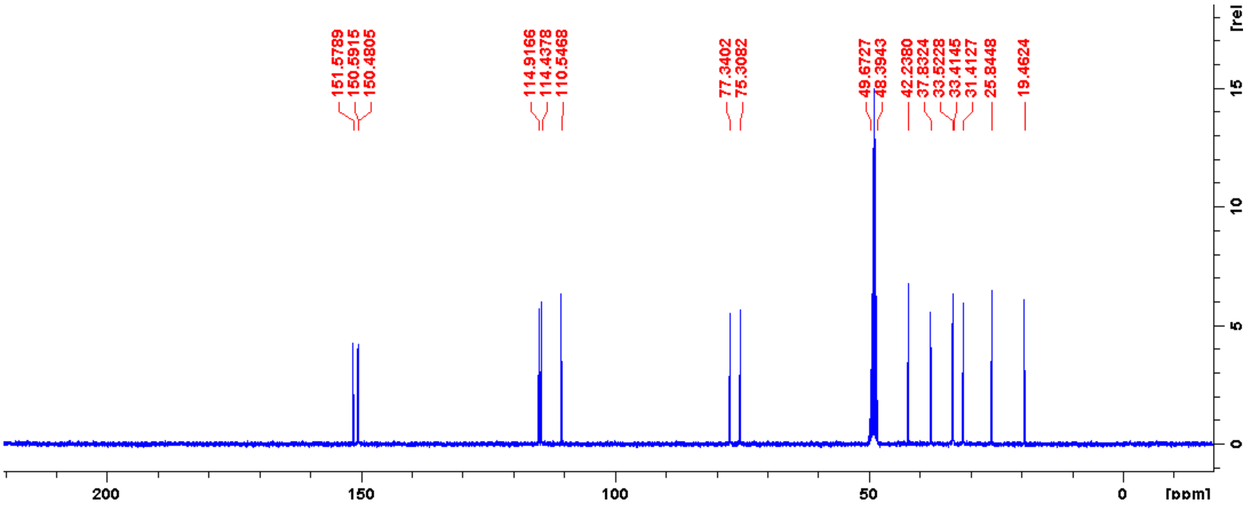

Figure S27. ^{1}H-^{1}H COSY spectrum of Compound **4** in CD_3OD.

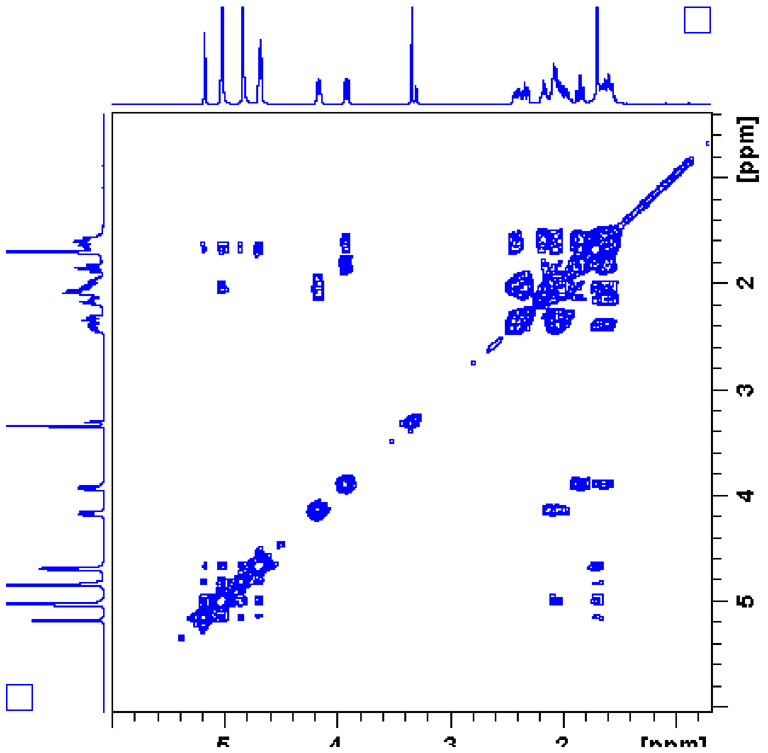

Figure S28. gHSQC spectrum of Compound **4** in CD_3OD.

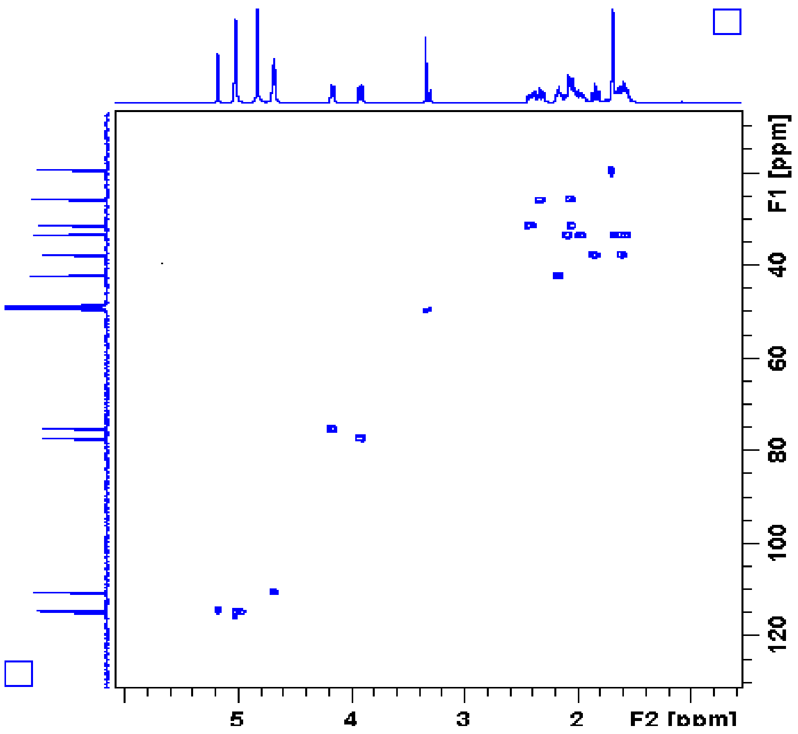

Figure S29. gHMBC spectrum of Compound **4** in CD_3OD.

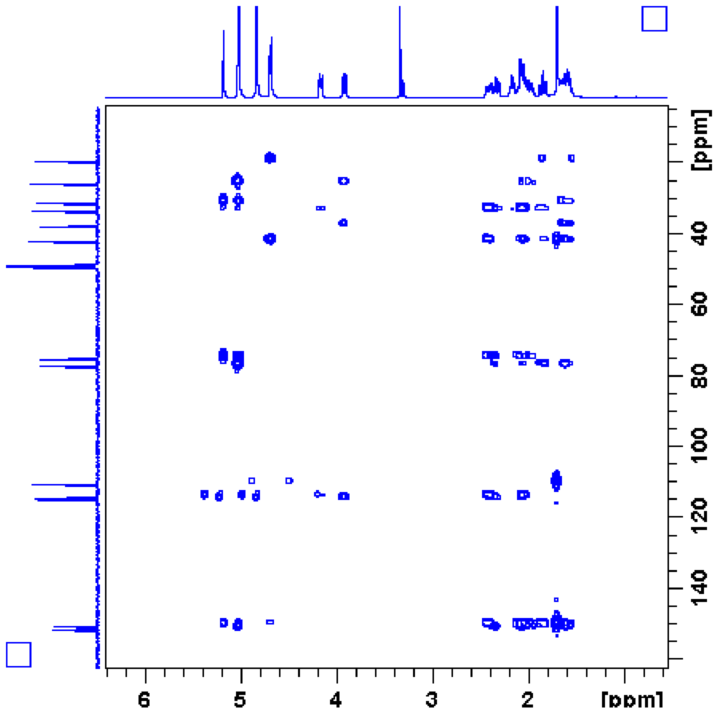

Figure S30. NOESY spectrum of Compound **4** in CD_3OD.

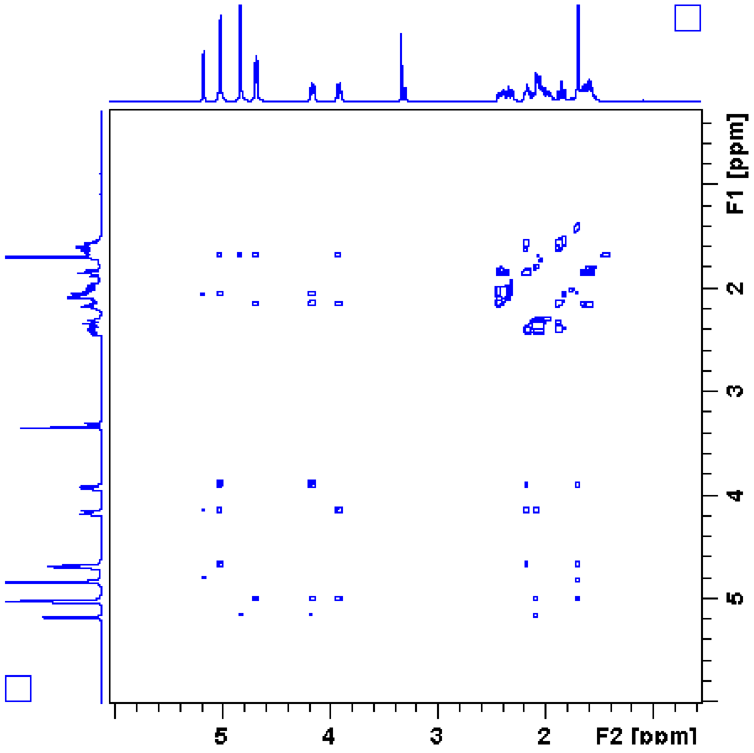

Figure S31. HRESIMS spectrum of Compound **4**.

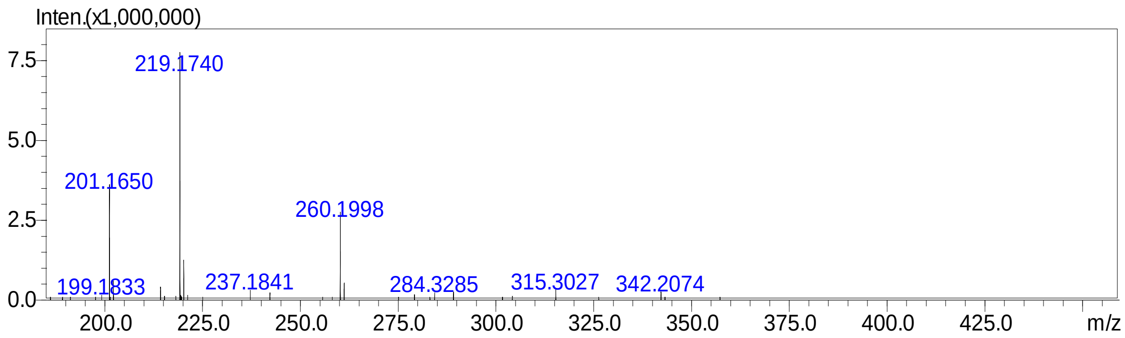

Figure S32. ^{1}H-NMR spectrum of Compound **5** in $CDCl_3$.

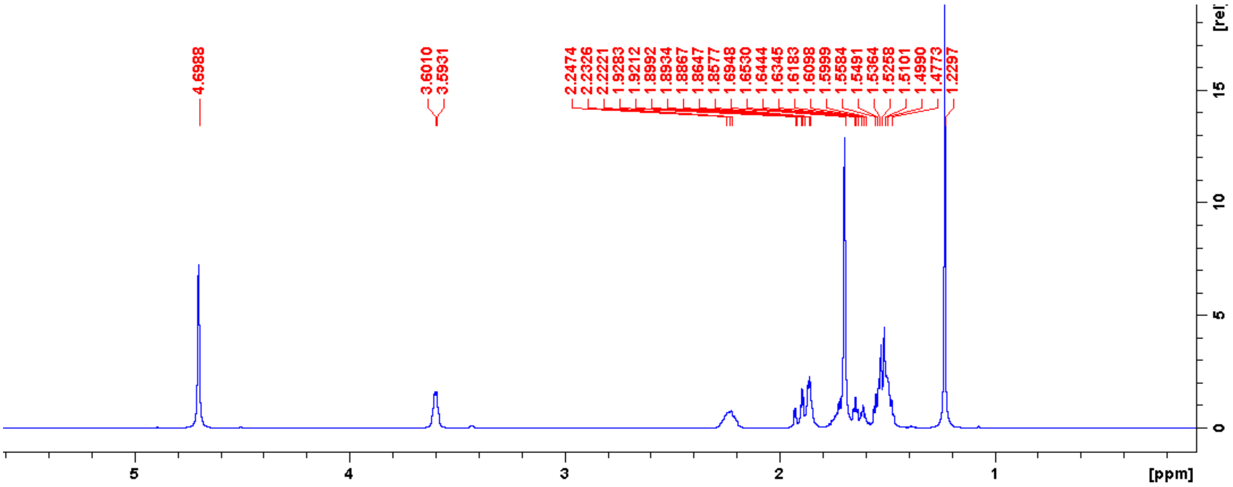

Figure S33. ^{13}C-NMR spectrum of Compound **5** in $CDCl_3$.

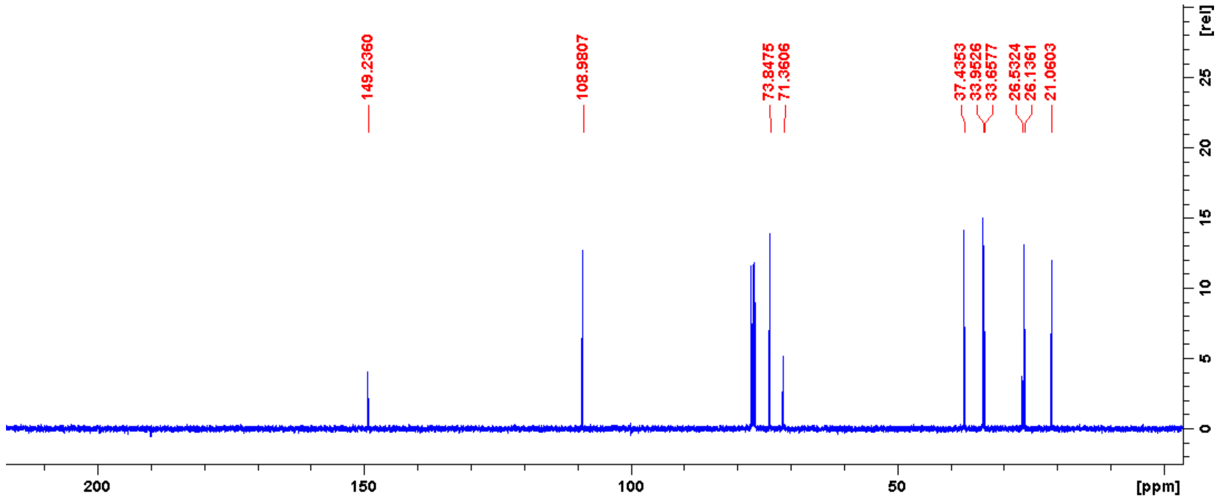

Figure S34. ^{1}H-^{1}H COSY spectrum of Compound **5** in $CDCl_3$.

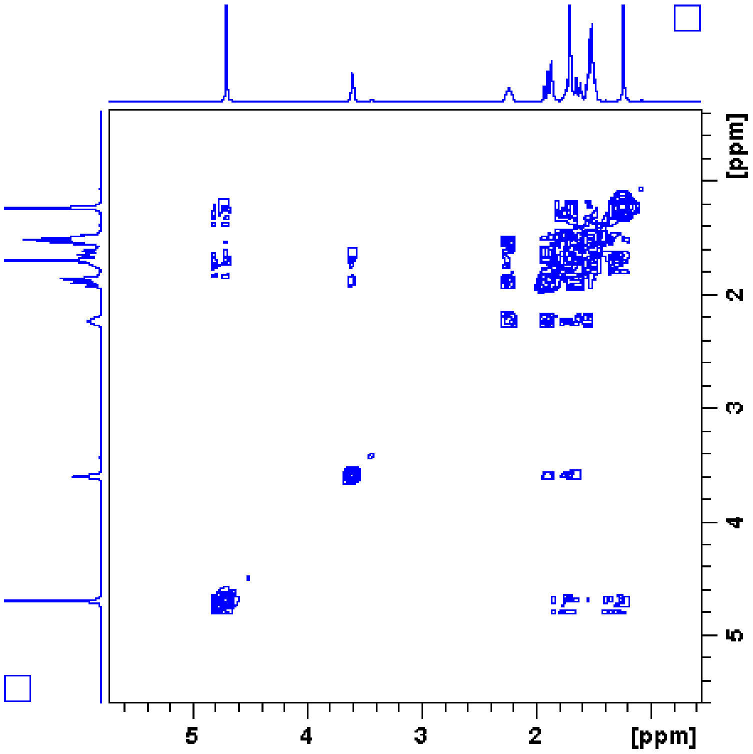

Figure S35. gHSQC spectrum of Compound **5** in $CDCl_3$.

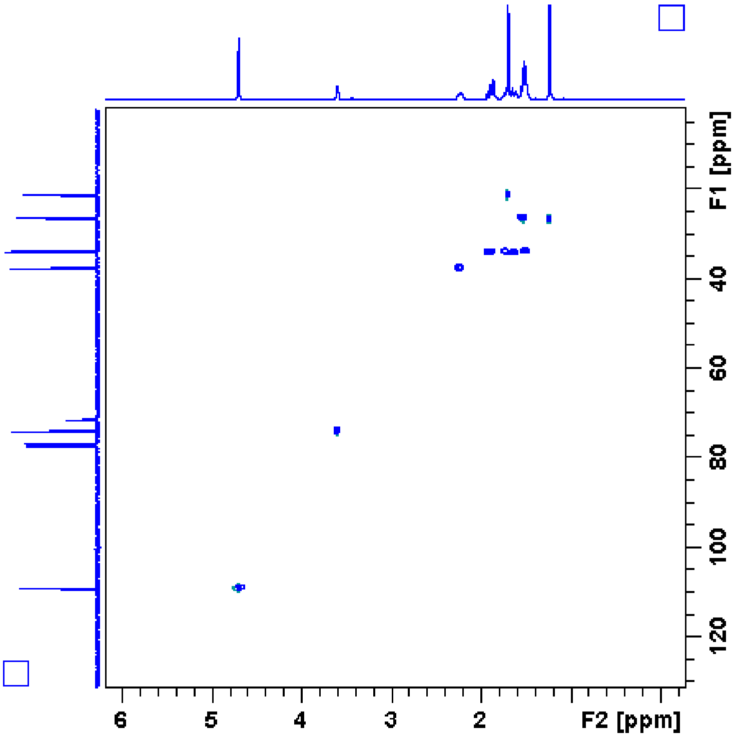

Figure S36. gHMBC spectrum of Compound **5** in $CDCl_3$.

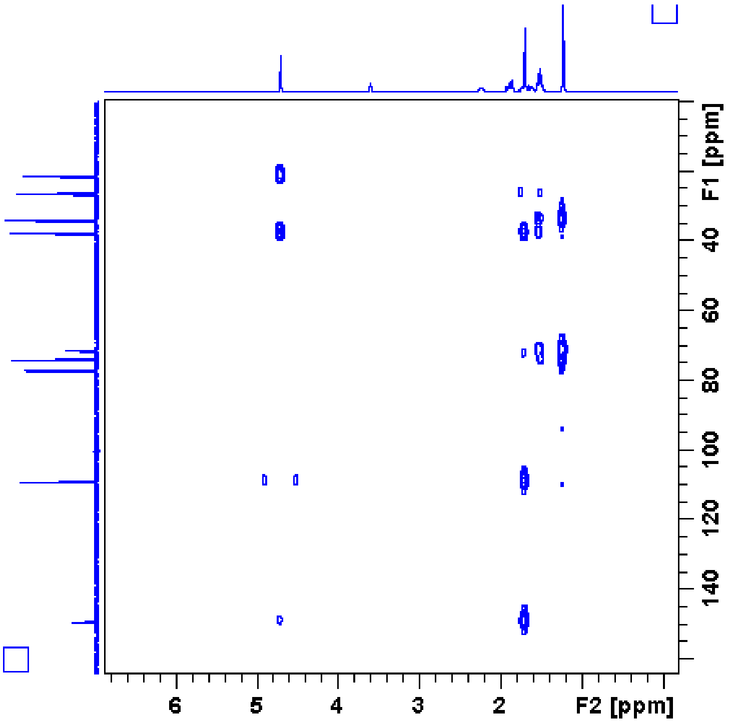

Figure S37. NOESY spectrum of Compound **5** in $CDCl_3$.

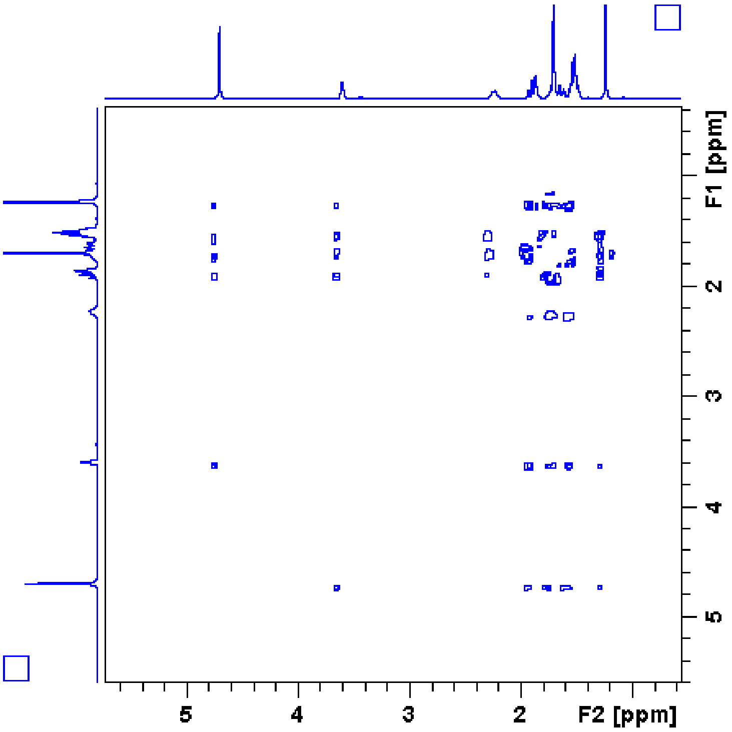

Figure S38. HRESIMS spectrum of Compound **5**.

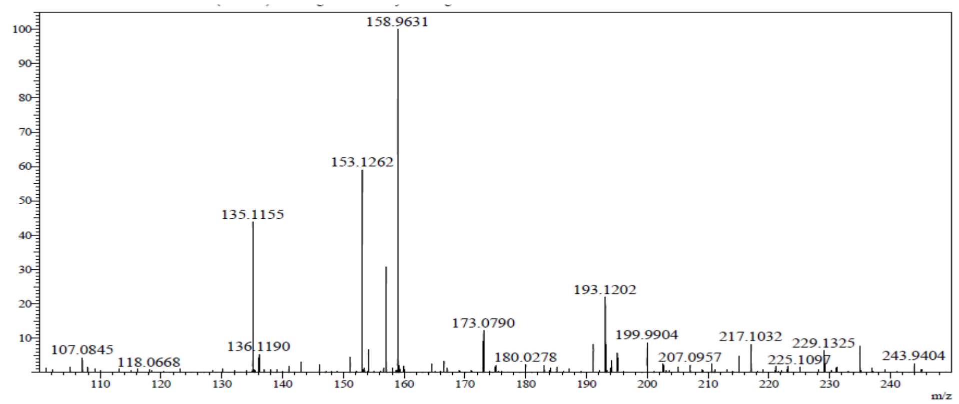

Supplement: Supplementary file 1 [file molecules-19-04326-s001.pdf]
